# Qualitative Conceptual Content Analysis of COVID-19 Vaccine Administration Error Inquiries

**DOI:** 10.3390/vaccines11020254

**Published:** 2023-01-24

**Authors:** Elisha Hall, Solomon Odafe, Joseph Madden, Sarah Schillie

**Affiliations:** 1 Communication and Education Branch, Immunization Services Division, National Center for Immunization and Respiratory Diseases, Centers for Disease Control and Prevention, 1600 Clifton Rd, MS A-19, Atlanta, GA 30329-4027, USA; 2 ASPPH/CDC Public Health Fellow (Embedded in Communication and Education Branch, Immunization Services Division, National Center for Immunization and Respiratory Diseases), Centers for Disease Control and Prevention, 1600 Clifton Rd, MS A-19, Atlanta, GA 30329-4027, USA

**Keywords:** COVID-19 vaccine, inquiries, errors

## Abstract

The launch of the COVID-19 vaccination program was the largest vaccination campaign in U.S. history, with an unprecedented demand for vaccine and new vaccination providers, warranting significant education and communication efforts. NIP-INFO (nipinfo@cdc.gov) is the Centers for Disease Control and Prevention’s (CDC’s) immunization inquiry response service, and it receives inquiries for COVID-19 and routine non-COVID vaccines. A qualitative analysis of NIP-INFO’s content was performed to better characterize and understand some of the knowledge gaps and reasons that COVID-19 vaccine administration errors occur. A total of 734 COVID-19 vaccine administration error inquiries were received between January 2021 and April 2022. The most frequent inquiries related to storage (*n* = 191; 26.0%), incorrect dosage or product (*n* = 190; 25.9%), unauthorized age group (*n* = 108; 14.7%), and schedule (*n* = 105; 14.3%). Training and communication strategies are imperative to ensure proper vaccine administration and build and maintain vaccine confidence.

## 1. Introduction

On 11 December 2020, the United States (U.S.) Food and Drug Administration (FDA) issued an Emergency Use Authorization (EUA) for use of the first COVID-19 vaccine in the United States, Pfizer-BioNTech, for people ages 16 years and older [1]. This EUA was followed by interim recommendations by the CDC’s Advisory Committee on Immunization Practices (ACIP). The Moderna COVID-19 vaccine was authorized soon after, followed by the Janssen COVID-19 vaccine in February 2021 [2,3]. Since this time, indications for vaccination have expanded to include younger age groups and booster doses, as well as the addition of the Novavax COVID-19 vaccine and multiple vaccine formulations or presentations. Some vaccines have been licensed in specific age groups under a Biologics License Application (BLA).

With the launch of the largest vaccination campaign in U.S. history, a surge in the vaccination provider workforce, and frequent updates to vaccination guidance, opportunities for vaccine administration errors existed [4,5]. The National Coordinating Council for Medication Error Reporting and Prevention defines a medication error as “any preventable event that may cause or lead to inappropriate medication use or patient harm while the medication is in the control of the health care professional, patient, or consumer [6]”. Medication errors are a leading cause of injury and avoidable harm in health care systems [7]. Limited studies have been conducted on COVID-19 vaccine administration errors; however, analyses of data from the Vaccine Adverse Event Reporting System (VAERS) indicate vaccine administration errors were the most common adverse events reported in the months following the authorization of COVID-19 vaccines for children and adolescents [8,9].

NIP-INFO (nipinfo@cdc.gov) is the Centers for Disease Control and Prevention’s (CDC’s) immunization inquiry response service. Public health providers, health care professionals, and the general public can submit clinically or programmatically relevant inquiries via email. NIP-INFO staff, including physicians, nurses, and health educators, respond to inquiries based on CDC or ACIP guidance. Although NIP-INFO inquiries likely represent only a small proportion of vaccine administration errors that occur, a qualitative analysis of their content can help to better characterize knowledge gaps and reasons why COVID-19 vaccine administration errors occur, providing the opportunity to improve communication and training materials. This study aims to describe health care provider knowledge gaps that led to COVID-19 vaccine administration errors.

## 2. Materials and Methods

This qualitative study used a conceptual content analysis design, specifically using manifest analysis, to describe the existence and frequency of concepts related to errors using a surface-level, descriptive approach [10,11]. This activity was reviewed by The National Center for Immunization and Respiratory Diseases’ human subjects contact reviewer and determined not to meet the definition of research, as defined in 46.102(l), and therefore, did not require institutional review board (IRB) review.

### 2.1. Data Collection

Data from 1 December 2020–30 April 2022 were exported into Microsoft Excel from the NIP-INFO email box using Microsoft Access. Data are regularly coded for vaccine type and cleaned by trained staff using a codebook containing high-level topics and definitions. A subset of inquiries was identified relating to the topics COVID-19 vaccine and vaccine administration errors. This sub-set of inquiries was disaggregated for inquiries with multiple questions in a single message (i.e., if an inquiry discussed multiple errors affecting multiple recipients, these errors were disaggregated to be coded appropriately).

Data were classified by inquirer type, including public health providers (i.e., public health clinics, state or county health departments, U.S. military, public schools), private providers (i.e., private clinics or providers, colleges or universities, pharmacies), unspecified type of provider, or general public (i.e., non-provider).

### 2.2. Data Analysis

Data were analyzed through manifest content analysis, which describes what is occurring on the surface (i.e., what inquirers actually say, staying close to the inquiry text) without identifying a deeper meaning. Two abstractors (E.H., J.M.) went through stages of:

(1) Decontextualization (identification of meaning units, i.e., coding): Abstractors read all emails to immerse themselves in the data. A codebook was developed, primarily deductively, using a priori codes based on the typical categorization of COVID-19 vaccine administration errors in the CDC’s Interim Clinical Considerations for COVID-19 Vaccines, and inductively, using email text to identify emerging codes [12]. Parent (main or superordinate) and child (subordinate) codes were identified. The text was divided into meaning units, and each meaning unit was coded separately using this codebook.

(2) Recontextualization (comparison of codes to original data): The codes were reviewed against the original emails and in relation to the study’s purpose. For intercoder agreement and reliability, abstractors completed the analysis independently and then discussed uncertainties and disagreements in coding and interpretation and reached consensus.

(3) Categorization: Codes with similar conceptual content were grouped together into subcategories. These categories were further condensed into one set of final categories that were internally homogenous and externally heterogeneous.

(4) Compilation: Conclusions were drawn regarding final categories through exploring the characteristics of each category, contextualizing categories in the context of the COVID-19 pandemic and existing research on routine vaccine administration errors, and making inferences about the findings.

Additionally, inquiry frequencies were calculated by error category and subcategory and source of the inquiry.

### 2.3. Data Visualization

Data were loaded into Microsoft PowerBI to produce the visualization used in this paper and to analyze trends.

## 3. Results

**Figure 1:**
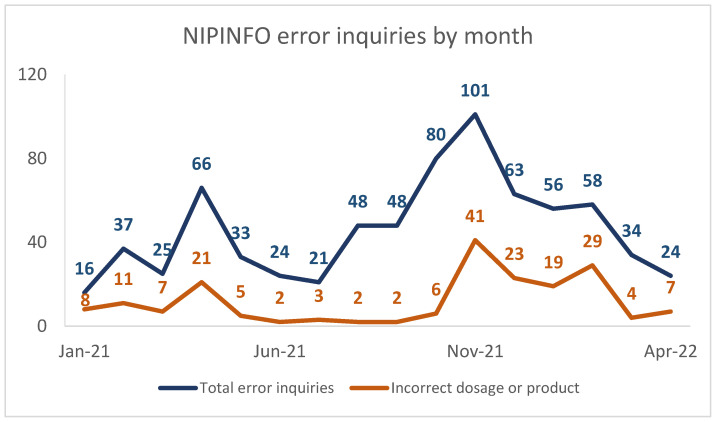
NIPINFO COVID-19 vaccine administration error inquiries by month, including total inquiries and incorrect dosage or product inquiries.

There were a total of 10,162 inquiries between 18 January 2021 through 30 April 2022 (no COVID-19 inquiries were received in December 2020). Of these, 734 were COVID-19 vaccine administration error inquiries (received 18 January 2021 through 29 April 2022), and these inquiries peaked in November 2021 (Figure 1). Leading up to and including this peak, there had been several updates to vaccine recommendations within a short period of time (Table 1). The most common source of inquiries was public health providers (*n* = 283; 38.6%), followed by private providers (*n* = 215; 29.3%), the general public (*n* = 141; 19.2%), and unspecified provider type (*n* = 95; 12.9%). Six categories and 16 subcategories of vaccine errors were derived from content analysis (Table 2).

### 3.1. Storage

Inquirers most frequently asked for guidance on vaccine storage errors (*n* = 191; 26.0%), including the administration of vaccine past the printed expiration date (the final day vaccine can be administered, as defined by the manufacturer; *n* = 52), past the beyond-use date (BUD) (the final day the vaccine can be administered, as defined by the provider when they transition vaccine to a different storage state or alter vaccine for use, i.e., puncture the vial stopper or dilute vaccine; *n* = 83), or after a temperature excursion (inappropriate storage conditions; *n* = 56).

Administering vaccine past the BUD was the most frequent storage error. This was most often related to not checking the BUD prior to vaccinating. Some providers did not understand the difference between a BUD and the expiration date, and that when deciding between the two, they should use whichever date comes first: “An 8-year-old patient received the correct Pfizer product on 1/25/2022 but the BUD was 1/21/2022 with an expiration date 1/31/2022”.

Practices described that led to temperature excursions included storage in inappropriate units, such as dormitory-style units, lack of temperature monitoring, lack of documentation of temperatures, and not checking the BUD prior to vaccination (e.g., a vial exceeded the time allowed in the freezer, but was not moved to the refrigerator). Despite new ultra-cold storage requirements with some COVID-19 vaccines, errors related to ultra-cold storage were rarely reported.

Storage errors often affected multiple vaccine recipients. Large-scale errors with hundreds or thousands of recipients occurred. In many cases, inquirers were state or jurisdictional staff who identified issues with storage units, temperature monitoring devices, or temperature records during a site visit.

### 3.2. Incorrect Dosage or Product

Incorrect dosage or product errors were the second-most frequent error inquiry (*n* = 190; 25.9%). Providers reported administering doses of vaccine that were both lower and higher than those authorized for the recipient’s age, but more often, the former (*n* = 151; 79.5%). The reported causes for lower-than-authorized dosages changed over the course of the analytic period. Early in the analytic period, providers reported basic vaccine administration issues not specifically related to COVID-19 vaccines that caused the vaccine to leak out (e.g., needle malfunction, the syringe breaking, the needle becoming disconnected from syringe, the patient moving or wiggling, etc.).

“A nurse was administering a Moderna COVID vaccine. As they were injecting the vaccine into the muscle, they noticed liquid coming out of the injection site, while the needle was still inserted in the patient’s muscle. They checked to see if the liquid was coming from the needle/hub area and found nothing coming from that area. It is unknown exactly how much of the vaccine was administered and how much was running down the patient’s arm”.

Later in the analytic period the cause was more often characterized as confusion between the more complex COVID-19 vaccine recommendations and products (e.g., confusing the amount of diluent needed between different products, the dosage between primary and booster doses, the dosage or product for different age groups, etc.).

“If [an] immunocompromised patient received the wrong dosage when given their 3rd Moderna vaccine should they repeat the 3rd dose? Patient received 0.25 mL Moderna as the 3rd dose but is immunocompromised and should have received 0.5 mL”.

Inquirers most often reported the causes for higher-than-authorized dosages being no or too little diluent, administering a multidose vial as if it were a single dose vial, or confusing the dosage or formulation.

The frequency of inquiries regarding incorrect dosage or product peaked first in April 2021, when states ended phased allocation to their populations and opened up vaccination to all eligible individuals, and generally remained low until November 2021, when the first formulation for children ages 5 through 11 years was authorized, and again in January 2022, when a third primary dose was authorized for immunocompromised children.

Public providers (providers representing state, local, or federal governments) were the most common subtype of inquirer in this category (*n* = 64; 33.7%).

### 3.3. Unauthorized Age Group

Inquirers also reported administering vaccine to unauthorized age groups (*n* = 108; 14.7%). This included administering vaccine to age groups that were not yet authorized for any COVID-19 vaccine and to age groups who were not authorized to receive a particular COVID-19 vaccine product. The most frequent error in this category was the administration of the Moderna COVID-19 vaccine to adolescents age 16 through 17 years prior to its authorization for this age group. This generally occurred when the provider intended to administer Pfizer, but used the wrong vaccine:

“We had a 16-year-old female who inadvertently received the Moderna vaccine for her first COVID-19 shot. Would this be considered a valid dose? Would we give her the second shot with the Moderna vaccine or should we restart the series with the Pfizer vaccine? If we use Pfizer, how long should we wait and would she need two doses?”

Overall, this inquiry type trended downward from January 2021–April 2022. This aligns with FDA’s authorization of COVID-19 vaccines to younger age groups over time, which made it increasingly unlikely for age-related errors to occur. When segmenting by age group, the common trend is an increase in errors up until the point when a certain age group was authorized to receive the COVID-19 vaccine, and then a steady decline afterwards. For the age groups of 16–17 years and 12–15 years, there are multiple peaks which correspond with the release of guidance regarding booster doses. Inquiries for unauthorized administration in people ages 5–11 years and under 5 years did not begin until August 2021 and January 2022, respectively, while inquiries concerning unauthorized administration in people over age 12 years have remained consistent since January 2021.

### 3.4. Schedule

Schedule errors (*n* = 105; 14.3%) most often included violation of the authorized interval between doses (i.e., primary series interval, booster interval, or interval after passive antibody products [when this recommendation was applicable]) (*n* = 45; 42.9%). Other schedule errors included using a mixed primary series, dispensing more total doses than authorized, or other incorrect product errors, such as administering the COVID-19 vaccine when the patient was scheduled for the influenza or shingles vaccination. Schedule inquiries were most often reported with mRNA COVID-19 vaccines (*n* = 80; 76.2%), which have more doses in the recommended scheduled and are the most widely used, compared to the Janssen COVID-19 vaccine, which is a single-dose primary series. Schedule errors demonstrated a relatively flat and consistent trend throughout the study period, peaking during September through November 2021, during which time there were several updates to the CDC’s recommendations. In September 2021, the first COVID-19 booster dose was authorized; booster recommendations subsequently underwent multiple changes over this time period (multiple booster products with different schedules, homologous and heterologous use, and high-risk population recommendations followed by universal recommendations).

Few inquirers described how scheduling errors occurred. Those who did indicated that the vaccination history was not properly verified. In these cases, providers did not verify the patient’s vaccination record card, medical record, or record in their state’s Immunization Information System (IIS). One inquirer that accepted a verbal report of vaccination history states that the patient “asked for a Moderna covid vaccine and did not tell us about the previous Pfizer vaccine. We administered the 1st Moderna vaccine”. Another that did not check records until after administration describes, “I have a patient who was screened prior to vaccination and he stated he had received no other vaccines. We proceeded with the 1st Moderna dose on 7/29. When we went to do our reporting and send the vaccine info in, unfortunately, we found out that he had received the J&J vaccine back in March”.

### 3.5. Preparation and Administration

Error inquiries concerning the preparation and physical administration of the COVID-19 vaccine (*n* = 71; 9.7%) most often included the incorrect site/route (*n* = 37; 52.1%) or incorrect diluent (*n* = 21; 29.6%). Other preparation and administration errors did not fall into one distinct subcategory (*n* = 13; 18.3%), such as withdrawing more doses than authorized from a multidose vial, using a potentially contaminated needle, using a single-dose diluent vial multiple times, and errors with documentation.

Errors involving the incorrect site/route most often involved both incorrect site and incorrect route. Inquirers described either administering or receiving a dose at the incorrect site (shoulder, bicep, triceps, or generally above or below the deltoid without further specification) and being concerned that this resulted in subcutaneous administration. Some inquirers indicated the vaccine provider pinched their skin to administer their dose. Most were concerned about the implications for protection against COVID-19: “I’m hoping you can provide some guidance; I received my second Moderna vaccine yesterday. I am concerned about incorrect administration. The nurse injected distal to my deltoid, plus she significantly pinched up my skin prior to injection. I really cannot tell if she injected into a muscle… I am concerned this could lead to inadequate immunity. I am a physician with significant patient contact daily, so my exposure risk to COVID is high. Do you have any guidance? Should the vaccine be readministered?”

The incorrect diluent was also identified as a preparation error with the Pfizer-BioNTech COVID-19 vaccine. The diluents used instead of manufacturer-supplied non-bacteriostatic 0.9% sodium chloride (normal saline) most often included sterile water or bacteriostatic saline.

## 4. Discussion

Vaccination remains one of the most effective public health interventions for preventing and controlling infectious diseases [22,23,24,25,26]. However, its continued success depends on the quality of the processes involved in vaccine administration [27,28]. During the time period of December 2020−April 2021, there were 31 updates to COVID-19 vaccination guidance and the release of 6 new COVID-19 vaccine products, adding significant complexity to recommendations over time for busy healthcare providers already receiving a plethora of information (e.g., information regarding quarantine, isolation, masking, ventilation, etc.). Our study highlights the challenges of communicating complex and frequently changing clinical immunization guidance. These findings suggest that there is a need to simplify and improve communication to reduce vaccine administration errors.

Storage errors accounted for approximately one-fourth of the error inquiries received, making it the most common error type identified in this analysis. A lack of understanding storage recommendations, particularly the difference between the BUD and expiration date, contributed to many of these errors. The expiration date is determined by the manufacturer and is the date after which the potency and safety of a vaccine can no longer be guaranteed [29]. In addition to the expiration date, some vaccines, such as reconstituted vaccines, vaccines supplied in multidose vials, or vaccines that have different temperature requirements for storage and administration, have a BUD that could be in days or hours [30]. The BUD supersedes, but should never exceed, the manufacturer’s expiration date; vaccines should not be used after the BUD. For example, the Moderna COVID-19 vaccine can be stored between −50 °C and −15 °C (−58 °F and 5 °F) until the expiration date, but stored between 2 °C and 8 °C (36 °F and 46 °F) for up to 30 days, up to 24 h at room temperature, or up to 12 h after the vial is punctured [31]. The Pfizer-BioNTech and Novavax COVID-19 vaccines have similar variations in BUD, depending on storage and handling conditions [32,33]. Storage errors, including administering a vaccine past the BUD, can lead to reduced vaccine potency and consequently, poor protection against disease [29]. Storage errors often affect large numbers of vaccine recipients, resulting in publicity and the need for a mass communication strategy.

Other errors that were a frequent source of inquiries included incorrect dosage or product (25.9% of inquiries), unauthorized age group (14.7% of inquiries), schedule errors (14.3% of inquiries), and preparation and administration errors, such as administration by the incorrect route or at an incorrect anatomic site (9.7% of inquiries). These errors can impact effectiveness and safety. Providers often described these errors occurring related to the complexity of products and vaccination schedule. Administration of a lower-than-authorized dosage, which may also occur when administering a vaccine product to an unauthorized age group (e.g., a pediatric product to an adult), may result in decreased vaccine effectiveness; therefore, the dose is recommended to be repeated. Decreased effectiveness may also result from inadvertent subcutaneous administration, as opposed to the recommended intramuscular administration, as subcutaneous fat has poor vascularity and slower antigen processing; however, there is a dearth of data on this error for COVID-19 vaccines. Very limited evidence suggests that an immune response is elicited with the subcutaneous administration of the Pfizer-BioNTech COVID-19 vaccine [34]. Unlike for some routinely recommended non-COVID-19 vaccines, due to lack of data, COVID-19 vaccine doses inadvertently administered subcutaneously are not recommended to be repeated. A higher-than-authorized dosage or a shortened interval may be associated with increased reactogenicity. For example, a higher risk of myocarditis has been observed with shorter intervals between mRNA and Novavax vaccine doses, most notably among adolescent and young adult men [12,35,36]. There are limited efficacy or effectiveness data on doses administered too closely together, but because of the risk of suboptimal immune response, it is recommended that a dose administered too early be repeated, as is recommended with routine vaccines. For COVID-19 vaccines, consideration is given to the risk for myocarditis when determining the spacing of the repeat dose.

Currently, there are only limited studies examining COVID-19 vaccine administration errors. A previous study that analyzed clinical inquiries at five COVID-19 vaccination centers in London reported mainly on patient factors such as allergies, immunosuppression, and compatibility of vaccines with other medications [37]. In April 2021, the Institute for Safe Medication Practices (ISMP) indicated that 20% of COVID-19 vaccine errors reported were due to lower- or higher-than-authorized dosage [38]. Some studies regarding the quality and service delivery of routine non-COVID-19 immunization processes have reported that challenges concerning the storage of vaccines remain a major limitation in the vaccine administration process [27,39,40,41,42]. A study on vaccine administration errors in a large academic center in the United States where over 1.4 million vaccines were administered reported that unfamiliar procedures and variations in procedures associated with vaccine administration increased the likelihood of vaccine administration errors [43].

A study that reviewed 4301 inquiries received between 2009 and 2011 by the Vaccine Advice for Clinicians Service (VACCSline), a service for health professionals concerning vaccinations within the Thames Valley Area of the United Kingdom, reported that 92% of all errors occurred during either vaccine selection and preparation or history checking and scheduling [44]. These findings have significant public health implications, as they show the critical process and knowledge gaps that may result in the inappropriate use of vaccines or patient harm [45]. Previously reported contributing factors to vaccine administration errors include inadequate human resources, frequent changes in guidelines, staff distraction, patient misidentification, misidentified products, the lack of familiarity with age-specific formulations, and using nonstandard or error-prone abbreviations [46,47]. Strategies to reduce vaccine administration errors include separating vaccines into bins or other containers according to type and formulation, preparing vaccines for one patient at a time in a no-interruption zone, and performing a triple-check before administering a vaccine. When an error does occur, the CDC recommends the recipient be informed about the error and CDC’s Interim Clinical Considerations for Use of COVID-19 Vaccines should be consulted for guidance regarding revaccination [12]. All COVID-19 vaccine administration errors, regardless of whether or not they were associated with an adverse event, are required to be reported to the Vaccine Adverse Event Reporting System (VAERS). To mitigate errors, the CDC regularly creates and delivers educational products to help providers more easily implement guidance, including: an extensive catalog of job aids to help providers store, handle, prepare, and administer vaccines correctly; regular local, state, and national educational presentations on clinical guidance; product-specific strategies to prevent errors; increased integration of visuals to facilitate understanding of complex recommendations; and generic educational products and training for general vaccination concepts. CDC also reviews error inquiries to better understand knowledge gaps and improve communication and education products for healthcare providers.

### Study Strengths and Limitations

Although NIP-INFO receives inquiries from across the United States and from a variety of inquirers (e.g., health department staff, physicians, nurses, pharmacists, and patients), inquiries received by NIP-INFO likely only represent a small proportion of COVID-19 vaccine administration errors. Providers are not required to report errors to NIP-INFO, but rather they contact NIP-INFO when seeking guidance for managing an error. A single inquiry received by NIP-INFO can reflect an error, such as a temperature excursion, affecting multiple vaccine recipients. Furthermore, some errors may go unrecognized, and not all inquiries received by the CDC are routed to NIP-INFO. Given the nature of the inquiry system, we were unable to validate that the errors actually occurred. As such, this analysis is not intended to be generalizable, nor to quantify COVID-19 vaccine administration errors, but rather to understand the errors that occur to guide future training and communication efforts.

## 5. Conclusions

Proper COVID-19 vaccine administration is necessary to achieve optimal vaccine-induced immune response, avoid safety implications, and assure confidence in the COVID-19 vaccination program and vaccines in general. Vaccine administration errors have the potential of creating mistrust and eroding vaccine confidence. This analysis highlights common COVID-19 vaccine administration errors and knowledge gaps that lead to those errors. Knowledge gaps exist for both routine vaccine recommendations (e.g., storage and administration best practices) and COVID-19 vaccine-specific recommendations (e.g., products, dosage, schedule).

The complexity of different COVID-19 vaccine products and frequently changing guidance have generated concern, with an ensuing ethical responsibility to simplify products, dosing, and labels, and subsequently, our communication concerning them, to reduce errors. Although the schedule for COVID-19 vaccination has been simplified over time, it remains complicated, raising an ethical need to balance the science (which might support different intervals for different doses or products) with implementation to simplify the schedule and reduce errors.

Upstream solutions to reduce schedule and product complexity may be more complicated to execute but have the potential to achieve significant and long-term impact in regards to preventing errors. Downstream solutions to educate providers and address identified knowledge gaps could focus on simple messaging for: general vaccine storage and administration (e.g., BUD, expiration, storage units, temperature monitoring, vaccination site and route, needle size, etc.) and COVID-19 vaccine recommendations (products, dosage, and schedule). A needs assessment for specific populations of healthcare providers can be conducted to better identify optimal content delivery (e.g., videos, fact sheets, infographics, microtraining) and promotion strategies for educational material. Future research could examine other intervention elements that could supplement education and communication to impact provider practices and reduce vaccine administration errors.

## Figures and Tables

**Table 1:** CDC COVID-19 vaccine recommendations published by month, December 2020–April 2022.

Month, Year	CDC Recommendation
December 2020	Pfizer-BioNTech COVID-19 vaccine primary series is recommended for eligible people age 16 years and older (eligibility based on phased allocation criteria) [13,14]. Moderna COVID-19 vaccine primary series is recommended for eligible people age 18 years and older (eligibility based on phased allocation criteria) [13,15].
February 2021	Janssen COVID-19 vaccine primary series is recommended for eligible people age 18 years and older (eligibility based on phased allocation criteria) [13,16].
March 2021	Phased allocation is lifted, and COVID-19 primary series vaccination using an age-appropriate vaccine is recommended for all people age 16 years and older.
April 2021	The use of the Janssen COVID-19 vaccine is paused while reviewing data involving cases of cerebral venous sinus thrombosis (CVST) with thrombocytopenia. The pause of the Janssen COVID-19 vaccine is lifted, and the FDA adds a warning for the rare occurrence of adverse events following the use of the Janssen COVID-19 vaccine [17].
May 2021	Pfizer-BioNTech COVID-19 vaccine primary series recommendation is expanded to people age 12–15 years [18].
August 2021	A third (i.e., “additional”) primary dose is recommended after 2 doses of an age-appropriate mRNA COVID-19 vaccine (Moderna or Pfizer-BioNTech) for people age 12 years and older who are moderately or severely immunocompromised [19].
September 2021	Pfizer-BioNTech COVID-19 vaccine booster dose is recommended in specific populations; authorized for homologous use only [19].
October 2021	Pfizer-BioNTech COVID-19 vaccine booster dose authorization is expanded for both homologous and heterologous use [19]. Janssen and Moderna COVID-19 vaccine booster doses are recommended in specific populations; authorized for homologous and heterologous use [19].
November 2021	Booster dose recommendations are expanded to all people age 18 years and older. Pfizer-BioNTech COVID-19 vaccine primary series recommendation is expanded to children age 5–11 years [20].
December 2021	Booster dose recommendations are expanded to all people age 18 years and older. Booster dose recommendations are expanded to all people age 16–17 years. mRNA COVID-19 vaccines are preferentially recommended over the Janssen COVID-19 vaccine in all persons due to the rare risk of thrombosis with thrombocytopenia syndrome [21].
January 2022	Booster dose recommendations are expanded to all people age 12–15 years. A third (i.e., “additional”) primary dose is recommended after 2 doses of the Pfizer-BioNTech COVID-19 vaccine for people age 5–11 years who are moderately or severely immunocompromised. An additional dose is recommended after 1 dose of the Janssen COVID-19 vaccine for people age 18 years and older who are moderately or severely immunocompromised.
March 2022	mRNA COVID-19 vaccines are recommended for use as a booster dose in specific populations as a second booster dose.

**Table 2:** Categories of COVID-19 vaccine administration errors.

Error Categories	Error Subcategories	Frequency n (%)
Storage	Expired by expiration date Past beyond-use date Temperature excursion	191 (26.0%)
Incorrect dosage or product	Higher-than-authorized dosage Lower-than-authorized dosage	190 (25.9%)
Unauthorized age group	Younger than age 5 years Age 5 through 11 years Age 12 through 15 years Age 16 through 17 years	108 (14.7%)
Schedule	Interval error Mixed primary series More doses than authorized Other incorrect product error	105 (14.3%)
Preparation and administration	Incorrect site or route Incorrect diluent Other preparation or administration error	71 (9.7%)
Multiple errors	N/A	69 (9.4%)

## Data Availability

Data included in this paper is not available for sharing due to privacy concerns.

## References

[1] U.S. Food and Drug Administration (2020). FDA Takes Key Action in Fight Against COVID-19 By Issuing Emergency Use Authorization for First COVID-19 Vaccine.

[2] U.S. Food and Drug Administration (2020). FDA Takes Additional Action in Fight Against COVID-19 By Issuing Emergency Use Authorization for Second COVID-19 Vaccine.

[3] U.S. Food and Drug Administration (2021). FDA Issues Emergency Use Authorization for Third COVID-19 Vaccine.

[4] U.S. Department of Health and Human Services (2021). Eighth Amendment to Declaration Under the Public Readiness and Emergency Preparedness Act for Medical Countermeasures Against COVID-19.

[5] U.S. Department of Health and Human Services (2021). Ninth Amendment to Declaration Under the Public Readiness and Emergency Preparedness Act for Medical Countermeasures Against COVID-19.

[6] National Coordinating Council for Medication Error Reporting and Prevention. About Medication Errors. https://www.nccmerp.org/about-medication-errors.

[7] World Health Organization Patient Safety. https://www.who.int/news-room/fact-sheets/detail/patient-safety.

[8] Hause A.M., Baggs J., Marquez P., Myers T.R., Gee J., Su J.R., Zhang B., Thompson D., Shimabukuro T.T., Shay D.K. (2021). COVID-19 Vaccine Safety in Children Aged 5–11 Years—United States, November 3–December 19, 2021. Morb. Mortal. Wkly. Rep..

[9] Hause A.M., Baggs J., Marquez P., Abara W.E., Olubajo B., Myers T.R., Su J.R., Thompson D., Gee J., Shimabukuro T.T. (2022). Safety Monitoring of COVID-19 Vaccine Booster Doses Among Persons Aged 12–17 Years—United States, December 9, 2021–February 20, 2022. Morb. Mortal. Wkly. Rep..

[10] Bengtsson M. (2016). How to plan and perform a qualitative study using content analysis. Nurs. Open.

[11] Kleinheksel A.J., Rockich-Winston N., Tawfik H., Wyatt T.R. (2020). Demystifying Content Analysis. Am. J. Pharm. Educ..

[12] Centers for Disease Control and Prevention (CDC). Interim Clinical Considerations for Use of COVID-19 Vaccines Currently Approved or Authorized in the United States. https://www.cdc.gov/vaccines/covid-19/clinical-considerations/interim-considerations-us.html.

[13] Dooling K., McClung N., Chamberland M., Marin M., Wallace M., Bell B.P., Lee G.M., Talbot H.K., Romero J.R., Oliver S.E. (2020). The Advisory Committee on Immunization Practices’ Interim Recommendation for Allocating Initial Supplies of COVID-19 Vaccine—United States, 2020. Morb. Mortal. Wkly. Rep..

[14] Oliver S.E., Gargano J.W., Marin M., Wallace M., Curran K.G., Chamberland M., McClung N., Campos-Outcalt D., Morgan R.L., Mbaeyi S. (2020). The Advisory Committee on Immunization Practices’ Interim Recommendation for Use of Pfizer-BioNTech COVID-19 Vaccine—United States, December 2020. Morb. Mortal. Wkly. Rep..

[15] Oliver S.E., Gargano J.W., Marin M., Wallace M., Curran K.G., Chamberland M., McClung N., Campos-Outcalt D., Morgan R.L., Mbaeyi S. (2021). The Advisory Committee on Immunization Practices’ Interim Recommendation for Use of Moderna COVID-19 Vaccine—United States, December 2020. Morb. Mortal. Wkly. Rep..

[16] Oliver S.E., Gargano J.W., Scobie H., Wallace M., Hadler S.C., Leung J., Blain A.E., McClung N., Campos-Outcalt D., Morgan R.L. (2021). The Advisory Committee on Immunization Practices’ Interim Recommendation for Use of Janssen COVID-19 Vaccine—United States, February 2021. Morb. Mortal. Wkly. Rep..

[17] MacNeil J.R., Su J.R., Broder K.R., Guh A.Y., Gargano J.W., Wallace M., Hadler S.C., Scobie H.M., Blain A.E., Moulia D. (2021). Updated Recommendations from the Advisory Committee on Immunization Practices for Use of the Janssen (Johnson & Johnson) COVID-19 Vaccine After Reports of Thrombosis with Thrombocytopenia Syndrome Among Vaccine Recipients—United States, April 2021. Morb. Mortal. Wkly. Rep..

[18] Wallace M., Woodworth K.R., Gargano J.W., Scobie H.M., Blain A.E., Moulia D., Chamberland M., Reisman N., Hadler S.C., MacNeil J.R. (2021). The Advisory Committee on Immunization Practices’ Interim Recommendation for Use of Pfizer-BioNTech COVID-19 Vaccine in Adolescents Aged 12–15 Years—United States, May 2021. Morb. Mortal. Wkly. Rep..

[19] Mbaeyi S., Oliver S.E., Collins J.P., Godfrey M., Goswami N.D., Hadler S.C., Jones J., Moline H., Moulia D., Reddy S. (2021). The Advisory Committee on Immunization Practices’ Interim Recommendations for Additional Primary and Booster Doses of COVID-19 Vaccines—United States, 2021. Morb. Mortal. Wkly. Rep..

[20] Woodworth K.R., Moulia D., Collins J.P., Hadler S.C., Jones J., Reddy S., Chamberland M., Campos-Outcalt D., Morgan R.L., Brooks O. (2021). The Advisory Committee on Immunization Practices’ Interim Recommendation for Use of Pfizer-BioNTech COVID-19 Vaccine in Children Aged 5–11 Years—United States, November 2021. Morb. Mortal. Wkly. Rep..

[21] Oliver S.E., Wallace M., See I., Mbaeyi S., Godfrey M., Hadler S.C., Jatlaoui T.C., Twentyman E., Hughes M.M., Rao A.K. (2022). Use of the Janssen (Johnson & Johnson) COVID-19 Vaccine: Updated Interim Recommendations from the Advisory Committee on Immunization Practices—United States, December 2021. Morb. Mortal. Wkly. Rep..

[22] Kim H., Kim H.S., Kim H.M., Kim M.J., Kwon K.T., Cha H.H., Seong W.J. (2022). Impact of vaccination and the omicron variant on COVID-19 severity in pregnant women. Am. J. Infect. Control.

[23] Kirson N., Swallow E., Lu J., Foroughi C., Bookhart B., DeMartino J.K., Maynard J., Shivdasani Y., Eid D., Lefebvre P. (2022). Increasing COVID-19 vaccination in the United States: Projected impact on cases, hospitalizations, and deaths by age and racial group. Public Health.

[24] Orangi S., Ojal J., Brand S.P., Orlendo C., Kairu A., Aziza R., Ogero M., Agweyu A., Warimwe G.M., Uyoga S. (2022). Epidemiological impact and cost-effectiveness analysis of COVID-19 vaccination in Kenya. BMJ Glob. Health.

[25] Sanz-Leon P., Hamilton L.H.W., Raison S.J., Pan A.J.X., Stevenson N.J., Stuart R.M., Abeysuriya R.G., Kerr C.C., Lambert S.B., Roberts J.A. (2022). Modelling herd immunity requirements in Queensland: Impact of vaccination effectiveness, hesitancy and variants of SARS-CoV-2. Philos. Trans. R. Soc. A.

[26] Stepanova M., Lam B., Younossi E., Felix S., Ziayee M., Price J., Pham H., de Avila L., Terra K., Austin P. (2022). The impact of variants and vaccination on the mortality and resource utilization of hospitalized patients with COVID-19. BMC Infect Dis..

[27] Thielmann A., Viehmann A., Weltermann B.M. (2015). Effectiveness of a web-based education program to improve vaccine storage conditions in primary care (Keep Cool): Study protocol for a randomized controlled trial. Trials.

[28] Hampton L.M. (2020). Vaccine handling and administration errors should be addressed to improve vaccine program safety. Vaccine.

[29] CDC Storage and Handling. https://www2.cdc.gov/vaccines/ed/covid19/SHVA/20010.asp.

[30] CDC (2022). Vaccine Storage and Handling Toolkit.

[31] CDC (2022). Moderna COVID-19 Vaccine, Storage and Handling Summary.

[32] CDC Storage and Handling of Pfizer-BioNTech COVID-19 Vaccines. https://www.cdc.gov/vaccines/covid-19/info-by-product/pfizer/storage.html.

[33] CDC Storage and Handling of Novavax COVID-19 Vaccines. https://www.cdc.gov/vaccines/covid-19/info-by-product/novavax/storage.html.

[34] Friedensohn L., Zur M., Timofeyev M., Burshtein S., Michael Y.B., Fink N., Glassberg E. (2021). Sub-cutaneous Pfizer/BioNTech COVID-19 vaccine administration results in seroconversion among young adults. Vaccine.

[35] Buchan S.A., Seo C.Y., Johnson C., Alley S., Kwong J.C., Nasreen S., Calzavara A., Lu D., Harris T.M., Yu K. (2022). Epidemiology of Myocarditis and Pericarditis Following mRNA Vaccination by Vaccine Product, Schedule, and Interdose Interval Among Adolescents and Adults in Ontario, Canada. JAMA Netw. Open.

[36] Pillay J., Gaudet L., Wingert A., Bialy L., Mackie A.S., Paterson D.I., Hartling L. (2022). Incidence, risk factors, natural history, and hypothesised mechanisms of myocarditis and pericarditis following covid-19 vaccination: Living evidence syntheses and review. Bmj.

[37] Bassi S., Begum R., Onatade R., Olie C. (2022). Analysis of clinical enquiries received by five COVID-19 vaccination centres in the UK. Eur. J. Hosp. Pharm..

[38] Institute for Safe Medication Practices (ISMP) Any New Process Poses a Risk for Errors: Learning from 4 Months of Coronavirus Disease 2019 (COVID-19) Vaccinations. https://www.ismp.org/resources/any-new-process-poses-risk-errors-learning-4-months-coronavirus-disease-2019-covid-19.

[39] Chiodini J. (2019). Vaccine storage: A hot topic for cool practice. Travel Med. Infect. Dis..

[40] Bulula N., Mwiru D.P., Swalehe O., Thomas Mori A. (2020). Vaccine storage and distribution between expanded program on immunization and medical store department in Tanzania: A cost-minimization analysis. Vaccine.

[41] Leidner A.J., Lee C.E., Tippins A., Messonnier M.L., Stevenson J.M. (2020). Evaluation of non-continuous temperature monitoring practices for vaccine storage units: A Monte Carlo simulation study. J. Public Health.

[42] Thielmann A., Puth M.T., Weltermann B. (2020). Improving knowledge on vaccine storage management in general practices: Learning effectiveness of an online-based program. Vaccine.

[43] Reed L., Tarini B.A., Andreae M.C. (2019). Vaccine administration error rates at a large academic medical center and its affiliated clinics-Familiarity matters. Vaccine.

[44] Lang S., Ford K.J., John T., Pollard A.J., McCarthy N.D. (2014). Immunisation errors reported to a vaccine advice service: Intelligence to improve practice. Qual. Prim. Care.

[45] Hibbs B.F., Miller E.R., Shimabukuro T., Centers for Disease C. (2014). Prevention. Notes from the field: Rotavirus vaccine administration errors—United States, 2006–2013. Morb. Mortal. Wkly. Rep..

[46] CDC Vaccine Administration: Preventing Vaccine Administration Errors. https://www.cdc.gov/vaccines/hcp/admin/downloads/vaccine-administration-preventing-errors.pdf.

[47] Paparella S.F. (2015). Vaccine Errors: Understanding the Risks and the Responsibilities for Public Safety. J. Emerg. Nurs..

